# A novel GnRH antagonist protocol based on LH levels versus traditional flexible GnRH antagonist protocol in PCOS patients undergoing in vitro fertilization: study protocol for a randomized controlled, non-inferiority trial

**DOI:** 10.1186/s13063-022-06586-1

**Published:** 2022-08-13

**Authors:** Shan Liu, Ya-su Lv, Shuo Han, Minghui Liu, Shuai Ma, Haiying Ren, Yuan Li

**Affiliations:** Medical Center for Human Reproduction, Beijing Chao-Yang Hospital, Capital Medical University, Beijing, China

**Keywords:** IVF, PCOS, LH, Effectiveness, GnRH antagonist protocol

## Abstract

**Background:**

The gonadotropin-releasing hormone (GnRH) antagonist protocol is advantageous given that it can avoid severe ovarian hyperstimulation syndrome (OHSS), especially for patients with polycystic ovary syndrome (PCOS). Basic and clinical evidence has shown that a threshold of luteinizing hormone (LH) stimulation is required for adequate follicular development and oocyte maturation. Ultra-low or high levels of LH are detrimental to pregnancy outcomes. We previously demonstrated that LH could be an indicator for the timing and dosage of antagonist administration in a retrospective study.

**Methods/design:**

In this randomized, single-center, non-inferiority trial, we aim to test the hypothesis that there is no significant difference in cumulative ongoing pregnancy rates between PCOS patients stimulated with LH-based flexible protocol versus traditional flexible GnRH antagonist protocol. The primary efficacy endpoint will be the cumulative ongoing pregnancy rate per cycle. The secondary outcomes will be clinical pregnancy rate, cancelation rate, serious OHSS rate, and cost-efficiency. The cumulative ongoing pregnancy rate per cycle in PCOS women was 80%. Considering that a non-inferiority threshold should retain 80% of the clinical effect of a control treatment, a minimal clinical difference of 16% (two-sided: *α*, 2.5%; *β*, 20%) and a total of 196 patients were needed. Anticipating a 10% dropout rate, the total number of patients required was 216.

**Discussion:**

The results of this study will provide evidence for the efficacy and safety of the LH-based flexible GnRH antagonist protocol in PCOS patients. Moreover, it evaluates the cost-efficiency of both protocols.

**Trial registration:**

Chinese Clinical Trial Registry ChiCTR1800018129. Date assigned: 31 August 2018. Protocol version: 1.0 (18 July 2017)

**Supplementary Information:**

The online version contains supplementary material available at 10.1186/s13063-022-06586-1.

## Background

Polycystic ovary syndrome (PCOS) is the most common heterogeneous endocrine disorder of women and is characterized by a clustering of hyperandrogenism, hypersecretion of luteinizing hormone (LH), and hyperinsulinemia, affecting approximately 9–18% of reproductive-aged women depending on definitions and the populations studied [1, 2]. In these patients, poor quality of oocytes leads to lower fertilization, implantation, and pregnancy rates and a higher miscarriage rate and eventually results in ovarian hyperstimulation syndrome (OHSS) [3–5]. Over the years, gonadotropin-releasing hormone (GnRH) antagonist protocols came to be used more frequently in a wide range of patients, including patients with poor, normal, or high ovarian responses [6, 7]. GnRH antagonist binds competitively to the GnRH receptor of the pituitary gland, but it cannot stimulate the pituitary to release follicle-stimulating hormone (FSH) and LH. According to the timing of initiation for the GnRH antagonist, GnRH antagonist protocols are divided into two types [8]: (i) *fixed protocol*, wherein the GnRH antagonist is initiated at a fixed time, generally in the 5–6 days of ovarian stimulation, and (ii) *flexible protocol*, wherein the antagonist is administered every day when the leading follicles reached 14 mm. It was concluded that a GnRH antagonist protocol is better than a long agonist protocol to reduce the rate of severe OHSS, and there was no difference in ongoing pregnancy, live birth rate, and other indicators between the two protocols [9, 10]. One of the common clinical manifestations of PCOS is the difference in the range of LH production and the variability in the LH:FSH ratio. We first used the LH level as an indicator for the timing and dosage of antagonist supplementation in a previous retrospective study. The results showed that patients with a sustained low LH concentration (LHmax of < 4 IU/L) during ovarian stimulation might not require antagonist administration [11]. In fact, not all PCOS patients had a premature LH surge during ovarian stimulation. On the other hand, part of them will always show a low level of LH. For such patients, the question remains whether it is necessary to add antagonists. For patients with sustained low LH concentration, administration of antagonist, either in traditional fixed or flexible GnRH antagonist protocol, might lead to even lower LH activity. Basic and clinical evidence has indicated that a threshold of LH stimulation is required for adequate follicular development and oocyte maturation. An ultra-low level of LH would do harm to pregnancy outcomes [12]. Our previous retrospective study data indicated that LH-based protocols might improve reproductive outcomes compared with a GnRH agonist during ovarian stimulation in normal responders [11]. A randomized controlled trial conducted in normal responders also showed that the LH-based GnRH antagonist protocol was not inferior to the traditional flexible GnRH antagonist protocol in clinical efficacy, and it was more cost-effective, considering the cumulative ongoing pregnancy rate in the entire assisted reproductive technology (ART) cycle (our unpublished data). However, there are currently limited data for the administration of this protocol in PCOS patients. Therefore, a randomized controlled trial was performed to prospectively compare the efficacy, safety, and cost-effectiveness of the novel LH-based flexible GnRH antagonist protocol with a traditional flexible GnRH antagonist protocol during ovarian stimulation.

## Methods/design

### Study design and recruitment

This is a single-blind, non-inferiority, randomized controlled trial. Patients participating in the trial are undergoing their 1st or 2nd in vitro fertilization (IVF)/intracytoplasmic sperm injection (ICSI) cycle in Beijing Chao-Yang Hospital, Capital Medical University. The research project was approved by the Ethics Committee of Beijing Chao-Yang Hospital and was conducted in accordance with Good Clinical Practices. The study was registered on ClinicalTrials.gov (No: ChiCTR1800018129). Potentially eligible women will be given information about the study at their first consultation. Screening for eligibility will be performed by an authorized investigator on day 2 of the menstrual cycle, after obtaining informed consent from all eligible participants. The study is currently recruiting patients and is expected to be completed by 31 December 2022.

### Eligibility criteria

Women will be enrolled in the study if they fulfilled the following criteria:

- Inclusion criteria:
  1. Infertile women planning a first or second cycle of IVF/ICSI treatment
  2. Diagnosed with PCOS according to the Rotterdam consensus criteria: irregular menstrual cycle, presence of ≥ 12 antral follicles (≤ 9 mm) in each ovary and/or ovarian volume > 10 mL on transvaginal ultrasonographic scanning, and/or clinical/biochemical hyperandrogenism [13]
  3. Aged between 20 and 38 years old
- Exclusion criteria:
  1. A partner with azoospermia
  2. Recurrent miscarriage or chromosomal abnormality
  3. Uterine fibroids, Mullerian malformations, or adnexal (hydrosalpinx) abnormalities
  4. Clinically significant systemic disease or other endocrine disorders

### Randomization and blinding

Included patients participated in no more than two treatment cycles. Women meeting the inclusion criteria will be randomized according to an online central randomization database (www.medresman.org) in a 1:1 ratio on the day of stimulation. The online sequence is generated and input into the online central randomization system by the staff members who are not involved in enrolling subjects. It is not accessible to any investigators or study coordinators. If a subject fulfills the enrollment criteria, the authorized investigator will log in to the password-protected account to get the assignment. After randomization, patients will be blinded to group allocation and randomly assigned to one of two groups: the control group (traditional flexible GnRH antagonist protocol) or the experimental group (LH-based flexible GnRH antagonist protocol). The nature of the treatment interventions precludes blinding of treating physicians. However, this study will be blinded to embryologists, laboratory technicians, and nurses who conduct follow-ups until the completion of the statistical analysis of this study.

### Sample size calculation

According to the literature and data from our center, the cumulative ongoing pregnancy rate per cycle in women with PCOS in the control arm was around 80%. Considering that a non-inferiority threshold should retain 80% of the clinical effect of a control treatment, a minimum clinical difference of 16% (two-sided: *α*, 2.5%; *β*, 20%) and a total of 196 patients were needed. Considering a possible withdrawal rate of 10%, the total number of patients required will be 216.

### Interventions

#### Adherence

Participants are fully informed that the trial does not involve the increase of drug types, blood test, and patient visits, which is easy for them to accept. The duration of ovarian stimulation ranges between 8 and 11 days for most patients. It will be finished by one fixed clinician for each participant. The attending in charge system will improve the relationship between clinicians and participants. They can consult the clinician in the clinic at each visit about the treatment regimen and adverse effects if any. Besides, psychological phenomena (e.g., depression and anxiety) can be reduced to improve adherence.

#### Ovarian stimulation

The ovarian stimulation is initiated from days 2 to 3 of the menstrual cycle. Baseline pelvic ultrasound and serum E_2_, FSH, LH, progesterone (P), and beta-human chorionic gonadotropin (β-hCG) were measured. Recombinant FSH (rFSH; Gonal-f, Merck, Germany) at a dose of 150–300 IU/day is administered depending on patient age, body mass index (BMI), anti-Mullerian hormone (AMH), antral follicle count (AFC), and basal serum FSH concentration. Five days later, the doses are adjusted according to ultrasound examination, follicle development, and serum hormone levels.

For the traditional flexible GnRH antagonist protocol, GnRH antagonist (Cetrotide, 0.25 mg per day; Merck, Germany) is initiated and continued up until and including the day of trigger, once the E_2_ concentration is ≥ 300 pg/mL or the leading follicle reaches a size of 14 mm.

For the LH-based flexible GnRH antagonist protocol, the timing and dosage of antagonist administration are based on the serum LH levels from day 5 of ovarian stimulation. The blood test will be performed 3–4 times until trigger. No antagonist is administered if the LH level was ≤ 4 IU/L. 0.125 mg of cetrorelix acetate is administered daily if the LH concentration is > 4 IU/L and ≤ 6 IU/L, until the next blood test. 0.25 mg of cetrorelix acetate is administered daily if the LH concentration was > 6 IU/L and ≤ 10 IU/L, until the next blood test. If the LH concentration is > 10 IU/L and ≤ 15 IU/L, 0.375 mg of cetrorelix acetate is administered daily for 1 day. If the LH concentration is > 15 IU/L, 0.5 mg of cetrorelix acetate is administered for 1 day. The need for antagonist co-treatment is dependent on an LH concentration of > 4 IU/L until the day of trigger. After two or more follicles reach a diameter ≥ 18 mm, 0.2 mg triptorelin and 2000–3000 IU of hCG will be injected. Serum LH, E_2_, and P concentrations are measured.

#### Oocyte retrieval, in vitro fertilization, and embryo transfer

Transvaginal ultrasound-guided oocyte retrieval will be performed 36 h later. Retrieved oocytes are fertilized by either IVF or ICSI, depending on sperm quality. For participants receiving fresh embryo transfer (ET), transfer is performed with two of the highest quality embryos at the cleavage stage 3 days after oocyte retrieval. Luteal-phase support with vaginal progesterone gel (Crinone, Merck Serono) at a dose of 90 mg daily and oral dydrogesterone (Duphaston, Abbott) at a dose of 10 mg twice daily is started after oocyte retrieval and continued until the day of hCG testing. If pregnancy is achieved, luteal phase support will be continued until 10 weeks’ gestation. Frozen ET will be taken if patients are at risk of OHSS; have an unfavorable endometrium (endometrial thickness of ≤ 6 mm or ≥ 16 mm, cavity fluid, or endometrial polyps); or have a P concentration of ≥ 1.5 ng/mL on the day of hCG trigger. For frozen ET, the endometrium is prepared using either a natural cycle regimen or an artificial cycle regimen based on the physician’s decision. For the natural cycle regimen, luteal phase support is started from the ovulation day with oral dydrogesterone (10 mg twice daily). For the artificial cycle regimen, the endometrium is prepared with oral estradiol valerate at a dose of 6–8 mg daily which will be started on days 3–5 of the menstrual cycle. Vaginal progesterone gel (90 mg daily) and oral dydrogesterone (10 mg twice daily) are added when the endometrium thickness reaches 8 mm. If pregnancy is achieved, luteal phase support will be continued until 10 weeks’ gestation.

Concomitant treatments such as traditional Chinese medicine and acupuncture are not permitted during the trial.

### Outcome measures

The primary outcome will be the cumulative ongoing pregnancy rate resulting from one ART aspiration cycle, including fresh and FET cycles. Ongoing pregnancy is defined by the presence of a gestational sac with a fetal heartbeat after 12 weeks of gestation. The cycle in which no oocytes are retrieved or no embryo is available for transfer will be considered as not getting pregnant. Secondary outcomes will be high-quality embryo rate, clinical pregnancy rate, severe OHSS rate, and cancelation rate. Moreover, differences in cost-effectiveness and adverse events will be evaluated. Typically, a high-quality embryo is defined as an embryo with 7–10 cells and ≤ 25% fragmentation developed from 2PN embryos on day 3 after oocyte aspiration. The clinical pregnancy rate is defined as the presence of a gestational sac at 6–7 weeks of gestation when visualized by transvaginal ultrasound. Moderate OHSS is diagnosed when ovarian enlargement of > 5 cm and < 12 cm is observed and when ultrasonographic ascites are present with or without nausea, vomiting, and/or diarrhea. Severe OHSS is diagnosed when ovarian enlargement of ≥ 12 cm is observed and when there is clinical evidence of ascites and/or hydrothorax or breathing difficulties with or without hemoconcentration, severe hypoproteinemia, abnormal liver function, coagulation abnormalities, or diminished renal function.

### Cost-effectiveness analysis

For both strategies, costs in the stimulation phase and effectiveness will be calculated from the complete cases. At the time of pregnancy testing, participants will be questioned to determine the costs of drugs in the stimulation phase. Furthermore, ongoing pregnancy achieved after natural conception or via conception other than a regular IVF or ICSI treatment is not included in the analysis.

### Data collection

Data for both clinical and economic outcomes will be collected by using a standard case report form developed in the web-based data entry system. All data will be entered into the database twice by two different doctors at the termination of the study to prevent operational errors. Once a patient is enrolled or randomized, the investigator will make every reasonable effort to follow her for the entire study period. Cell phone numbers of the wife, husband, and another family member will all be recorded. The prespecified staff are responsible for the follow-up. During the trial, there will be several other methods to improve retention, such as scheduling appointments with the clinician and keeping in contact with the investigators through apps such as WeChat. The relevant description has been added to the manuscript.

### Data monitoring and safety

All data will be supervised by independent statisticians from Beijing Chao-Yang Hospital. All clinical research will be carried out as per the Good Clinical Practice (GCP) standards. The study site monitor will check the maintenance of trial-related source records, authenticity, and integrity of the data regularly to ensure adherence to the protocol, standard operating procedures, and applicable regulatory requirements. Adverse events (AEs) are defined as any undesirable event experienced by a subject during a clinical trial, regardless of whether the event is considered related to the intervention. Routine assessments for OHSS were performed on day 2 after oocyte retrieval in both groups. At all other times, OHSS will be evaluated and classified if symptoms are reported by the patient. A serious adverse event (SAE) is defined as any untoward medical occurrence or effect that results in death, is life-threatening (at the time of the event), requires hospitalization, prolongs an existing hospitalization, results in persistent or significant disability or incapacity, is a congenital anomaly or birth defect, and is a new event of the trial likely to affect the safety of the subjects such as an unexpected outcome of an adverse reaction. All AEs reported spontaneously by the subject or observed by the investigator or their staff will be recorded accurately in the eCRFs and will be followed up during the course of the AE until their resolution or for 2 weeks after the end of the trial. All SAEs will be reported to the investigators, discussed through a web-based AE reporting system, and will be reported to the Pharmaceuticals and Medical Devices Agency, if necessary. There will be an interim analysis. If the data show convincing evidence of harm, such as a high cancelation rate due to premature LH surge, the trial would be stopped by the investigators or the data monitoring committee.

Over the course of the trial, some tiny modifications, such as modification of the follow-up method from telephone to short message for some subjects, will not trigger a protocol amendment. However, protocol amendment will be performed if the most relevant and critical modifications that affect the trial design and data collection procedure occurred. A modified version, if applicable, will be submitted to the sponsor for funding, to the IRB for ethics approval, and to the data monitoring committee.

### Statistical analysis

Data analysis of this trial will follow the intention-to-treat principle, including a preliminary comparison of all randomized women in the two groups who did not drop out of the study. Differences between the groups will be compared between demographic variables and baseline information from before the start of the study. Continuous data with normal distribution will be estimated using a two-sample *t*-test, and the Mann-Whitney *U* test will be used for non-normally distributed data. Categorical data will be represented as frequency and percentage, and the differences in these variables will be assessed by the chi-square test or Fisher’s exact test. We will also compute the unadjusted risk ratio (RR) and its 95% confidence interval (CI). This test for non-inferiority will only be performed for the primary outcome; all other secondary outcomes will be tests of superiority. The primary outcome—cumulative ongoing pregnancy rate per cycle in women with PCOS in the control arm—was 80% in our center. Considering that a non-inferiority threshold should retain 80% of the clinical effect of a control treatment, the minimum clinical difference should be 16%. Noninferiority is declared if the upper limit of the two-sided 95% CI for the cumulative ongoing pregnancy rate in the study group does not exceed a relative margin of 16%, equivalent to a two-sided test with an *α* value of 2.5%. A two-sided *α* value of 0.05 is used for superiority testing.

In case of missing values of baseline characteristics, we will analyze them by excluding the missing values, then assign the missing values multiple times, and conduct subsequent analyses to estimate the robustness of the results. For follow-up loss and protocol violation, we will attempt sensitivity analysis to explore the influence of these factors on the trial results.

**Fig. 1:**
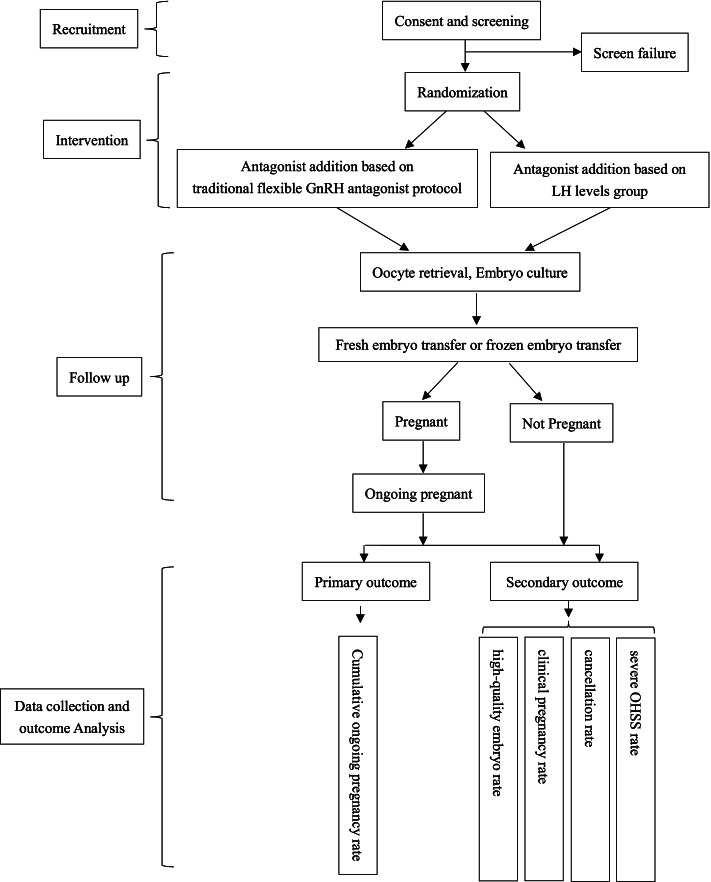
Flowchart per the checklist of Standard Protocol Items: Recommendations for Interventional Trials (SPIRIT) showing patient enrollment, allocation, treatment, and follow-up of participants

**Fig. 2:**
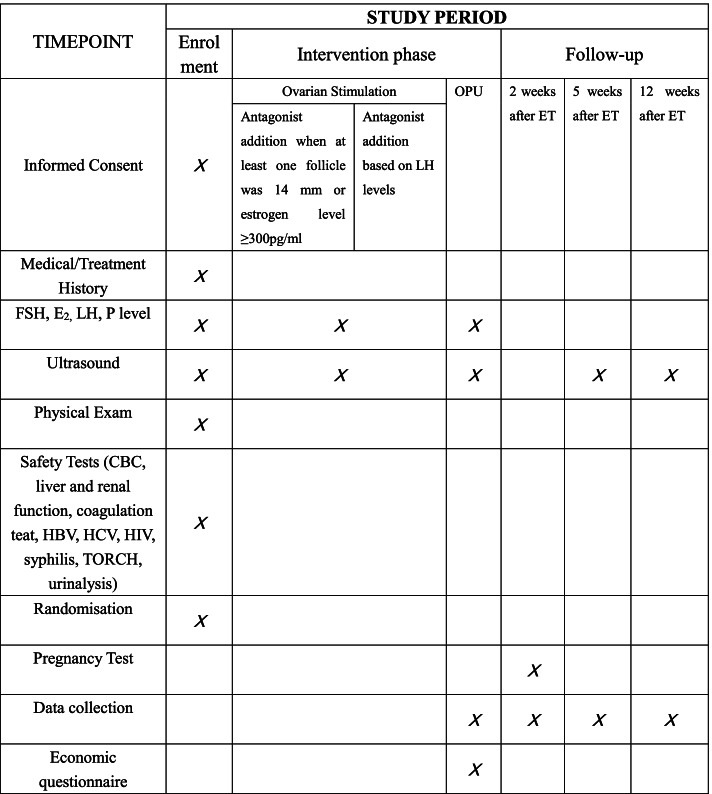
SPIRIT figure: the schedule of enrollment, interventions, and assessments

The flow chart of this study is given in Fig. 1. A schedule of enrollment, interventions, and assessment is provided in the Standard Protocol Items: Recommendations for Interventional Trials (SPIRIT) figure (Fig. 2), and the SPIRIT reporting guidelines are used as shown in Additional file 1 [14].

## Discussion

PCOS is a common reproductive endocrine disorder frequently associated with elevated endogenous LH secretion; however, the effects of LH are unpredictable. A study showed that the elevated basal day 2/3 LH levels and LH/FSH ratio do not impair the outcome of GnRH antagonist protocol-treated IVF/ICSI cycles in patients with PCOS [15], which illustrates that suppressing LH values before IVF is not clinically worthwhile for individual patient management. On the other hand, endogenous LH levels are not enough to fully support the development of follicles in some patients. This viewpoint is increasingly being adopted globally. In addition, previous studies indicated that profound LH suppression is detrimental for patients undergoing either GnRH agonist- or GnRH antagonist-treated cycles [16, 17]. The role of LH in PCOS has not been fully studied and seems to be exaggerated without adequate evidence.

Our retrospective cohort study showed that LH could be used as an indicator for the timing and dose of antagonist administration in the GnRH antagonist protocol [11]. We hope that the result will be applicable to a wider population, such as PCOS patients. The trial has a randomized, controlled design which should minimize bias. Data are being collected from a single study center with all investigators required to undertake mandatory training in the protocol, limiting the generalizability of the results. There are also some limitations: the study population is from China and further studies are necessary to verify our conclusions in other ethnic populations, given the genotype and phenotype variations of different ethnic groups. We believe that this proposed trial is unique in that it explores broader considerations, including both the effectiveness, safety, and cost of drugs.

## Trial status

The study was conceived and designed in 2017. Enrollment began in 2018 and is expected to end in December 2022. At the time of manuscript preparation, more than 180 subjects have been enrolled. Enrollment in this study was ongoing at the time of manuscript submission.

## Supplementary Information

**Additional file 1. **SPIRIT Checklist for *Trials*.

## Data Availability

The datasets generated during the current study are available from the corresponding author on reasonable request.

## Abbreviations

AFC: Antral follicle count
AMH: Anti-Müllerian hormone
ART: Assisted reproductive technology
BMI: Body mass index
DMC: Data Monitoring Committee
E2: Estradiol
eCRF: Electronic case report form
ET: Embryo transfer
FSH: Follicle-stimulating hormone
GCP: Good Clinical Practice
Gn: Gonadotropin
GnRH: Gonadotropin-releasing hormone
hCG: Human chorionic gonadotropin
ICSI: Intracytoplasmic sperm injection
IVF: In vitro fertilization
OHSS: Ovarian hyperstimulation syndrome
P: Progesterone
RCT: Randomized controlled trial
SAE: Serious adverse event
SPIRIT: Standard Protocol Items: Recommendations for Interventional Trials
